# Supplementary material for: Diversity and specificity of molecular functions in cyanobacterial symbionts
Source: Sci Rep. 2024 Aug 12;14:18658. doi: 10.1038/s41598-024-69215-8 (PMC11319675; doi:10.1038/s41598-024-69215-8)
Supplement: Supplementary file 1 — Supplementary Figures. [file 41598_2024_69215_MOESM1_ESM.docx]

**Figure S1:** Comparison of completeness and contamination estimates for eleven genomes that met quality filtering thresholds (>90% completeness; <5% contamination) with CheckM but not CheckM2 which were retained for analysis.

**Figure S2:** Frequency count of KEGG functions completeness score. Majority of detected functions occur in high completeness.


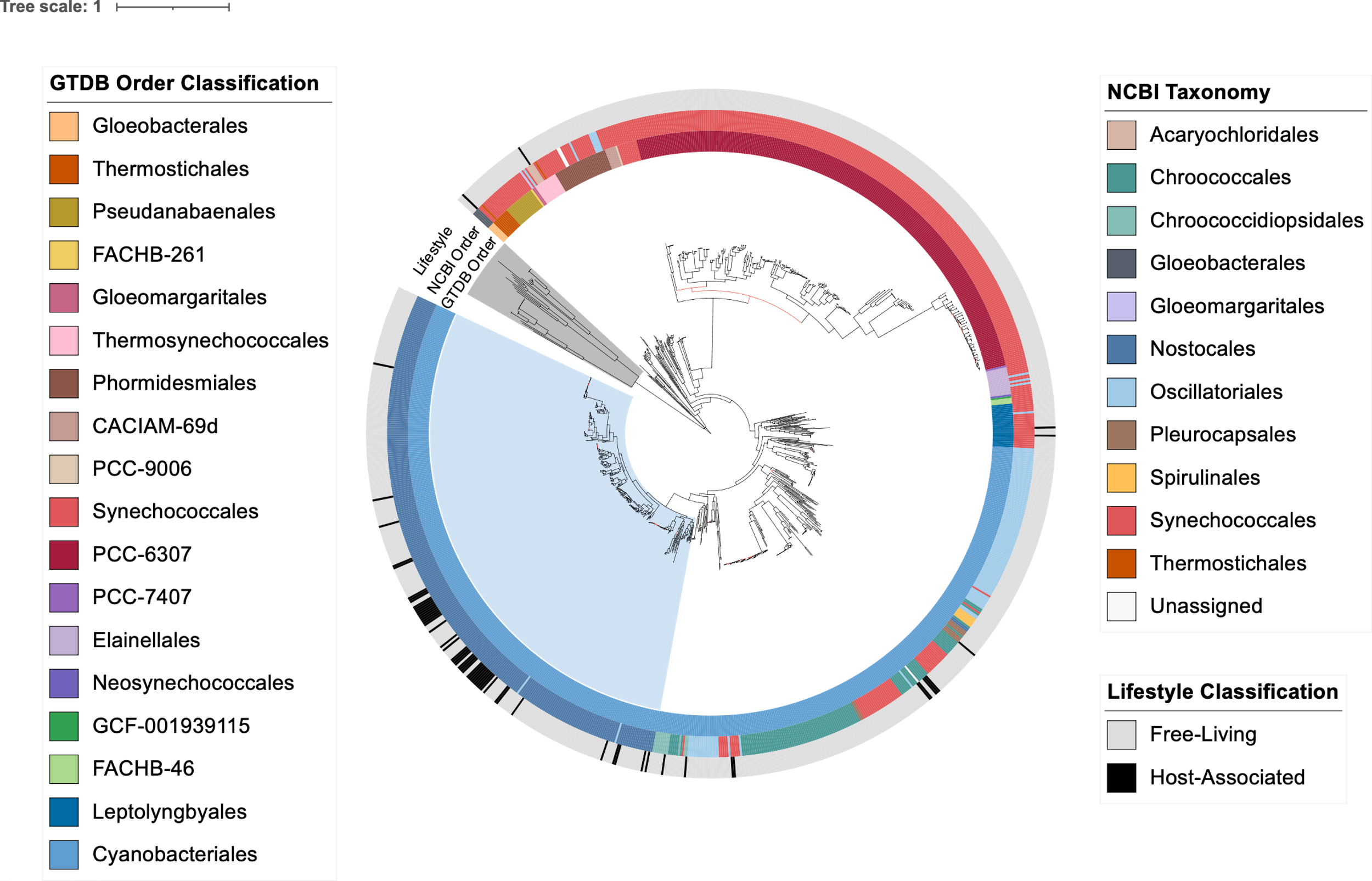


**Figure S3:**  Phylogeny generated using concatenated marker genes of genome sequences of strains from phylum Cyanobacteria, rooted with representatives of the sister group, Melainabacteria, with 1000 bootstraps. Branches with high bootstrap support (>80%) are shown with black. The outer annotation track depicts the lifestyle classification to highlight host-associated cyanobacterial symbionts. The inner annotation track depicts the classified taxonomic order assigned by GTDB, and the middle track depicts the NCBI taxonomic classification revealing non-monophyletic distribution of current orders across the phyla.

**Figure S4:** Phylogeny generated using concatenated marker genes of genome sequences of strains from phylum Cyanobacteria, rooted with representatives of the sister group, Melainabacteria, with 1000 bootstraps. Branches with high bootstrap support (>80%) are shown with black. From inside to outside annotation tracks depict the i) classified taxonomic order assigned by GTDB, ii) lifestyle classification to highlight host-associated cyanobacterial symbionts, and iii) remaining tracks depict the distribution of molecular functions found to be significantly associated with lifestyle classification. Remaining tracks depicted represent the following functions and processes (from inside to outside): (I) Photosystem II, (II) MEP-DOXP Pathway, (III) Methionine metabolism, (IV) Fe-Mn transporter, (V) F-type ATPase, (VI) NAD(P)H-quinone oxidoreductase, (VII) riboflavin biosynthesis, (VIII) sulfide oxidation, (IX) glucoamylase, (X) zeaxanthin diglucoside, (XI) urea transporters, (XII) leucine metabolism, (XIII) ferric iron substrate inding (AfuA), (XIV) cytochrome bd complex, (XV) formate metabolism, (XVI) cobalt transporters (CbiMQ), (XVII) gluconeogenesis, (XVIII) cobalt transporter (CorA), (XIX) D-galacturonate epimerase, (XX) nitrogen fixation. Permission for utilizing the KEGG database was obtained from Kanehisa laboratories^46^.

**Figure S5:** Phylogeny generated using concatenated marker genes of genome sequences of strains from phylum Cyanobacteria, rooted with representatives of the sister group, Melainabacteria, with 1000 bootstraps. Branches with high bootstrap support (>80%) are shown with black. From inside to outside annotation tracks depict the i) classified taxonomic order assigned by GTDB, ii) lifestyle classification to highlight host-associated cyanobacterial symbionts, and iii) remaining tracks depict the distribution of BGC groups generated through Louvain4 clustering found to be significantly impacted by lifestyle classification.

**Figure S6**: Counts of KEGGdecoder functions detected in Nostocaceae genomes to reveal ubiquitously occurring functions and metabolism pathways. Permission for utilizing the KEGG database was obtained from Kanehisa laboratories^46^.

**Figure S7:** Phylogeny generated using concatenated marker genes of genome sequences of strains from family Nostocaceae, rooted with representatives of the order Elainellales, with 1000 bootstraps. Genera with symbiotic lifestyles are highlighted. From inside to outside annotation tracks depict the i) isolation source, and ii) remaining tracks depict the distribution of molecular functions found to be significantly associated with isolation source. Remaining tracks depicted represent the following functions and processes (from inside to outside): (I) glucoamylase, (II) Fe-Mn transporter, (III) photosystem II, (IV) phenylalanine metabolism, (V) phosphonate transporters, (VI) chitinase, (VII) methionine metabolism, (VIII) sulfur dioxygenase, (IX) cytochrome bd complex. Permission for utilizing the KEGG database was obtained from Kanehisa laboratories^46^.


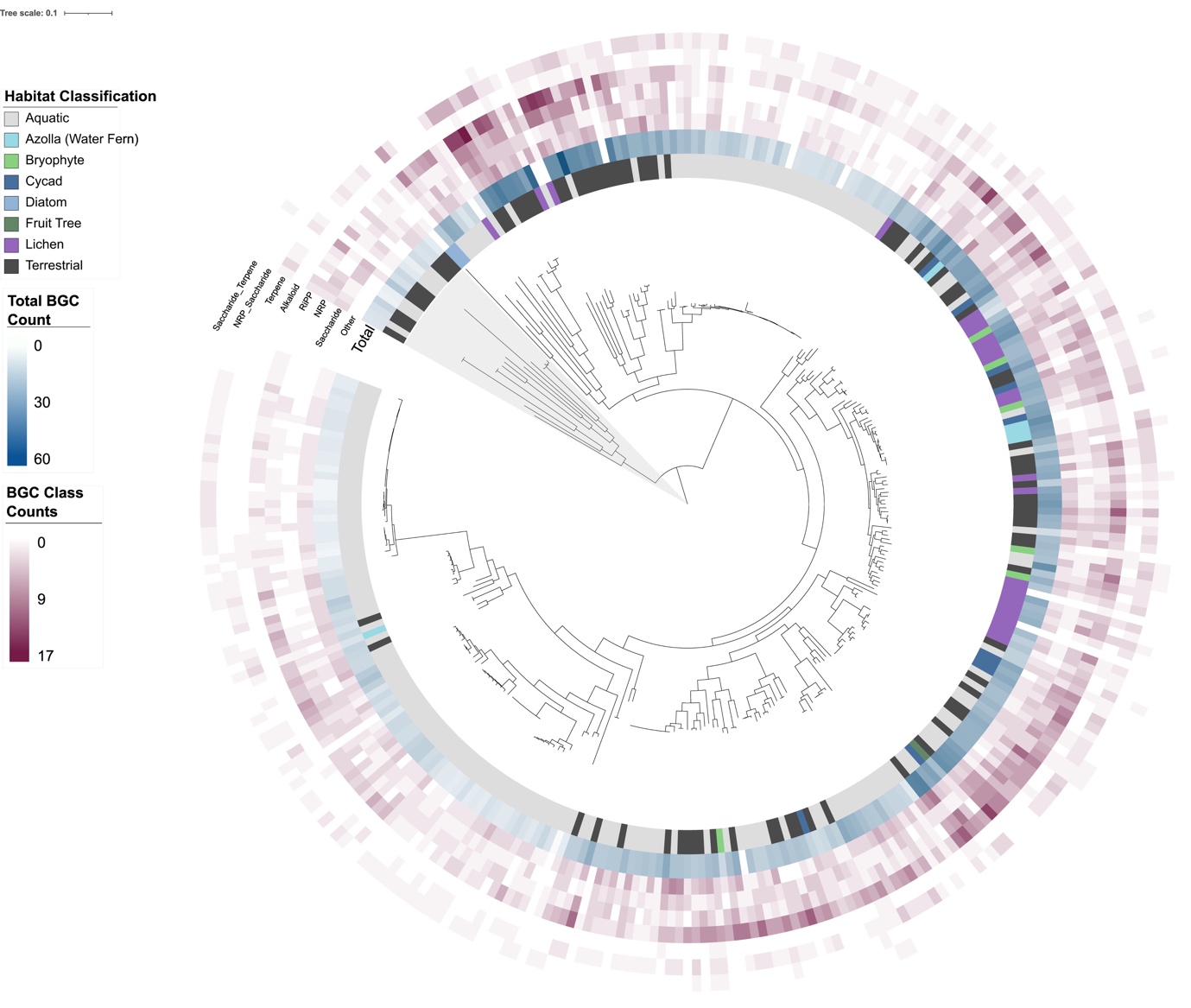


**Figure S8**: Phylogeny generated using concatenated marker genes of genome sequences of strains from family Nostocaceae, rooted with representatives of the order Elainellales (depicted with light grey colour blocking on branches), with 1000 bootstraps. Genera with symbiotic lifestyles are highlighted. From inside to outside annotation tracks depict the i) isolation source, and ii) remaining tracks depict the distribution of total counts and counts of individual biosynthetic gene clusters found to be significantly associated with isolation source.

**Figure S9:** Phylogeny generated using concatenated marker genes of genome sequences of strains from family Nostocaceae, rooted with representatives of the order Elainellales (depicted with light grey colour blocking on branches), with 1000 bootstraps. Genera with symbiotic lifestyles are highlighted. From inside to outside annotation tracks depict the i) isolation source, and ii) remaining tracks depict the distribution of BGC groups generated through Louvain4 clustering found to be significantly impacted by lifestyle classification.
